# The de Clérambault syndrome: More than just a delusional disorder?

**DOI:** 10.1192/j.eurpsy.2021.177

**Published:** 2021-08-13

**Authors:** A. Fiorillo

**Affiliations:** Department Of Psychiatry, University of Campania “L. Vanvitelli”, Naples, Italy

**Keywords:** Delusion, psychosis, affective symptoms

## Abstract

The de Clèrambault syndrome is a psychiatric condition characterized by the presence of a delusion in which the patient is convinced that another person has fallen in love with him or her. Patients usually believe that their lover is a person belonging to a higher social and economic class, or is already married, or even is imaginary or deceased person. In the majority of cases, the patients do not seek for psychiatric help, but usually is referred to the mental health care system due to behavioural consequences associated with the syndrome, including stalking behaviours (repetitive calling, unexpected visits or continuous attempts to send gifts or letters to the loved person). The name of the syndrome derives from the French psychiatrist Gaetan Gatian de Clerambault, who systematically described this syndrome in a series of patients. According to the modern classification systems, the syndrome is conceptualized as erotomanic subtype of the delusional disorder. However, the presence of delusions is not the only clinical feature of the syndrome. In fact, specific affective features are usually present, such as grandiosity, hypersexuality and promiscuity. Therefore, it has been argued that De Clèrambault syndrome should be considered as lying on the continuum of the spectrum of bipolar disorders. Those diagnostic uncertainties highlight the difficulties for clinicians to properly manage this syndrome and should represent a valid reason for rediscovering this almost neglected psychiatric syndrome.

**Disclosure:**

No significant relationships.

